# Newborn genetic screening for spinal muscular atrophy in the UK: The views of the general population

**DOI:** 10.1002/mgg3.353

**Published:** 2017-11-23

**Authors:** Felicity K. Boardman, Chloe Sadler, Philip J. Young

**Affiliations:** ^1^ Division of Health Sciences Warwick Medical School University of Warwick Coventry UK; ^2^ School of Life Sciences University of Warwick Coventry UK

**Keywords:** bloodspot, ethics, newborn genetic screening, social implications, spinal muscular atrophy

## Abstract

**Background:**

Spinal muscular atrophy (SMA) is an inherited neuromuscular disorder and a leading genetic cause of infant death worldwide. However, there is no routine screening program for SMA in the UK. Lack of treatments and the inability of screening tests to accurately predict disease severity are among the key reasons implementation of screening has faltered in the UK. With the recent release of the first therapy for SMA (Nusinersen), calls are being made for a reconsideration of this stance; however, very little is known about the views of the general public.

**Methods:**

An online survey was administered to 232 individuals with no prior relationship with SMA to assess their attitudes toward a newborn screening program for it. Results are compared with previously gathered data on the views of SMA‐affected families toward screening.

**Results:**

Eighty‐four percent of participants were in favor of newborn screening. Key reasons for support were a belief that it would lead to better healthcare and life expectancy for affected infants and facilitate informed decision‐making for future pregnancies. Key reasons for nonsupport were a belief in the potential for significant negative impact on the family unit in terms of bonding and stress.

**Conclusions:**

Public acceptability is a key component in the evaluation of any potential screening program in the UK. This study demonstrates that newborn screening for SMA is viewed largely positively by people unfamiliar with the condition. The importance of early identification overrode all other social and ethical concerns about screening for the majority of participants.

## 1 INTRODUCTION

With the development of technologies such as whole genome sequencing, population screening for increasing numbers of genetic disorders is now more feasible than ever before. While many of the genetic disorders for which screening could be introduced are considered rare, most have limited treatment options, a substantial impact on quality of life and unpredictable/variable trajectories (Rose, 2015). Genetic screening for such conditions, it has been argued, would allow carrier parents the option of avoiding the birth of an affected child (when carried out preconceptually or prenatally), or the timely introduction of therapies or clinical trial enrollment (when carried out on newborns). As well as earlier identification, newborn genetic screening is also associated with what has been termed ‘reproductive benefit’ (Bombard et al., 2009). That is, through their identification as carriers, parents (as well as their wider family) will be able to make informed decisions in relation to any subsequent pregnancies (Botkin & Rothwell, 2016). Moreover, some newborn genetic screening programs, for example, that for cystic fibrosis in the UK, are capable of not only identifying infants who have (or will develop) cystic fibrosis, but also those infants who are genetic carriers. Thus, the “reproductive benefit” of newborn screening may potentially also be conferred on the screened child.

Despite these possibilities for enhanced reproductive options and/or treatments, however, the introduction of newborn genetic screening for rare disorders is still somewhat controversial. This is particularly so when undertaken through whole genome sequencing techniques which have the capacity to generate large (and potentially unwieldy) volumes of data and within which the boundaries between pathogenic and nonpathogenic findings are not clear (Friedman, Goldenberg, Lister, Sénécal, & Vears, 2017). The presymptomatic genetic testing of children for incurable and/or late‐onset disorders, furthermore, has long been considered ethically problematic, primarily because the direct benefits to the child are limited (Schmidt et al., 2012), and indeed, there can be associated harms. These harms include the possibility of false positive/negative results, the impact of the results on the relationship between parent and child, the loss of disease‐free time and anxiety about the future (Tluczek, Orland, & Cavanagh, 2011). Indeed, asymptomatic children who are diagnosed with a late‐onset condition shortly after birth have been described by Timmermans & Buchbinder (2010) and Grob (2008) as existing in “liminal” state: neither fully ill nor fully well, with varying implications for roles, identities, and (familial) relationships.

Aside from the implications for the child, ethical concerns have also been raised around the possible limits on informed consent for expanded newborn genetic screening programs (Bailey, Skinner, Davis, Whitmarsh, & Powell, 2008; Taylor‐Phillips et al., 2014). Members of the general population approach genetic screening with little/no background knowledge of the condition for which they, or their child will be screened, and research has demonstrated that they are often entirely unprepared for a positive result (Dankert‐Roelse & Meerman, 1995; McClaren, Delatycki, Collins, Metcalfe, & Aitken, 2008). Indeed, it has been argued that in an age of expansive genomic screening, that notions of informed consent may have to be adapted (Himes et al., 2016; O'Neill, 2001; Rose, 2015).

With these benefits and concerns in mind, this paper considers the attitudes of members of the general public toward a potential newborn screening program for spinal muscular atrophy (SMA), a condition for which newborn screening is currently being considered, both in the UK and in the USA. While our previous study outlined the views of families living with SMA toward newborn screening (Boardman, Young, & Griffiths, 2017a,b,c), this present study contrasts these findings with the views of members of the general population who have no prior relationship with the condition. The analysis explores how far newborn genetic screening is accepted among its intended recipients, highlighting the degree to which prior knowledge of the condition being screened for is a significant factor in attitudes.

### 1.1 Spinal muscular atrophy and newborn genetic screening

Spinal muscular atrophy is a neurodegenerative disorder primarily resulting from the degradation of the alpha motor neurons which connect the spinal cord, resulting in progressive proximal muscle weakness (Munsat & Davies, 1992, 1996). Spinal muscular atrophy can be characterized into different types (I–IV) based on time of onset and achievement of motor milestones: Type I (severe, onset: <1 year); Type II (intermediate, onset: 7–18 months); Type III (mild, onset >2 years); and Type IV (mild, adult onset) (Lunn & Wang, 2008; Wang & Lunn, 2008).

There are currently numerous tests that can be used to diagnose SMA, including restriction fragment length polymorphism, multiplex ligation‐dependent probe amplification, and quantitative PCR (Ar Rochmah et al., 2017; Kato et al., 2015; Kesari, Mukherjee, & Mittal, 2003; Ogino, Leonard, Rennert, & Wilson, 2002; Ogino & Wilson, 2002; Xu, Ogino, Lip, Fang, & Wu, 2003). In addition, there is now a noninvasive prenatal diagnostic analysis that can diagnose SMA using blood from expecting mothers, although due to the nature of the analysis, this test can only be offered to mothers who already have a child diagnosed with SMA, making it an unsuitable screening tool (Parks et al., 2017).

As all forms of SMA are caused by mutations in the telomeric *survival motor neuron* (*SMN1*) gene (Lefebvre, Burglen, Frezal, Munnich, & Melki, 1998; Lefebvre et al., 1995, 1997), the second copy of SMN (centomeric *SMN*;* SMN2*) is an important disease‐modifying gene (Lefebvre et al., 1995). Indeed, *SMN2* copy number has been described by some researchers as being linked to disease severity in the majority of cases (with higher numbers of SMN2 copies being associated with milder pathology), although this approach has been somewhat controversial, and criticized for being overly simplistic without consideration of potential gene modifiers (Oprea et al., 2008). In practice, therefore geno‐ *and* phenotype data are taken into consideration when a symptomatic child is diagnosed with SMA (Gavrilov, Shi, Das, Gilliam, & Wang, 1998; Wadman et al., 2017; Wirth et al., 2006).

When SMA is diagnosed through a screening program, however, there are unique challenges. The combined lack of family history, together with a (potentially) asymptomatic infant may make an accurate prognosis for the child difficult (Prior et al., 2010). Indeed, a newborn screening program would lead to the identification of not only severely affected infants (those with type I SMA who are unlikely to live past 18 months), but also those who will go on to develop milder forms of the condition later in life (Prior et al., 2010: p;. 1613). Given the vast spectrum of presentations associated with SMA, a clear idea of the likely course of the disease—and critically, whether the child is expected to survive infancy—is of paramount importance to new parents. Inability to provide accurate information about type was indeed one of the key reasons that SMA screening was not introduced in the UK after the last public consultation (Cartwright, 2012).

Spinal muscular atrophy screening is currently in place in several countries internationally, with Qatar implementing compulsory premarital carrier screening, and countries, such as Australia and Israel, offering screening as part of state‐subsidized healthcare programs (Boardman et al., 2017b,c). Newborn screening for SMA has long been called for within the US; however, efforts to introduce it have been consistently thwarted by the absence of an effective therapy, a prerequisite for all new newborn screening programs (Swoboda, 2010). The licensing of Nusinersen (Spinraza) at the end of 2016, however, following promising results of phase I clinical trials (Chiriboga et al., 2017), dramatically shifted the landscape of SMA newborn screening. Nusinersen therapy (administered via repeated intrathecal injections) was demonstrated to have a significant impact on the motor function and survival of type I affected infants. As it has been demonstrated that Nusinersen therapy is most effective when administered in presymptomatic children (Bertini, Hwu, Reyna, Farwell, & De, 2017), the case for the introduction of state‐wide newborn screening to identify infants before they become symptomatic has been considerably strengthened in recent months. Indeed, newborn screening for SMA is currently under review for inclusion on the Federal Newborn Recommended Uniform Screening Panel and in July 2017, Missouri became the first state to mandate SMA newborn screening. Within the UK, newborn screening for SMA is considered periodically.

As well as the development of therapies, a small number of pilot studies have also been undertaken to demonstrate both the feasibility of a newborn screening program for SMA (Prior, 2010a,b,c; Prior et al., 2010), and limited number of studies have also explored attitudes toward such screening among parents (Rothwell, Anderson, Swoboda, Stark, & Botkin, 2013), the general public (Lin et al., 2016), and also families who live with SMA (Boardman et al., 2017b; Wood et al., 2014). This paper contributes to this increasingly important body of literature by exploring attitudes toward newborn screening for SMA among a large sample of people (*n* = 232) from the UK general population, and offers a comparison with our previous work with affected families.

## 2 METHODS AND MATERIALS

### 2.1 SMA screening survey and SMA newborn screening survey (UK)

The SMA Screening Survey (UK) was developed from qualitative interview data from interviews with people living with SMA or their families, as described elsewhere (Boardman et al., 2017a,b,c). For this study, questions regarding newborn screening were selected and reproduced in a shorter survey (UK NewGenPop Survey). Questions used to assess the demographics of respondents were either modified versions of, or directly taken from, questions used in the 2011 UK Census survey. As participants had no previous experience with SMA, key information about the condition, its inheritance and presentation was provided at the start of the survey. Ethical approval of the survey was awarded in July 2014 by the Biomedical and Scientific Research Ethics Committee.

### 2.2 Survey distribution

Quantitative data collection was carried out from January to May 2017. The UK newborn survey was only available online through the survey platform Qualtrics. Participants were invited to complete the survey if they were over the age of 18 and had no relationship with SMA. The survey was distributed through social networking pages (Facebook). All participants remained anonymous by distributing the survey link generated by the Qualtrics platform: (http://warwick.co1.qualtrics.com/jfe/form/SV_d1jLEuyn0o2 ha17). The link was reusable and was unable to track identifying information of respondents.

### 2.3 Statistical analysis

Basic descriptive analysis was performed to show the percentage of respondents associated with each demographic characteristic. Responses were stratified as follows: gender (male 1 vs. other 0); age (18–25 years 1 vs. ≥26 years 0); qualifications (degree 1 vs. other 0); religion (yes 1 vs. other 0). For responses to each question regarding newborn screening, data were stratified as either strongly agree/agree (1) or other (0). This allowed all positive views to be assessed in comparison to those with negative or uncertain views.

Percentages of respondents that answered either agree (1) or other (0) to questions regarding newborn screening were calculated and compared to the responses of people with SMA and their families using a chi‐square test.

Univariate binary logistic regression was performed to find associations between those that had ‘agree’ responses to each question regarding newborn screening and those who stated they would support a newborn genetic screening program for SMA. This allowed for the effect of each independent variable to be assessed. For those that had a significant association, multivariate binary logistic regression was performed to find questions independently associated with the test variable. Last, in order to assess bias among the demographics, forward multivariate binary logistic regression using the identified main drivers, age, gender, qualification, and religion as the independent variables was performed. During the course of the statistical analysis, any probability value (*p* value) of <.05 was considered significant. All statistical analysis was performed using IBM SPSS Statistics 24 software.

## 3 RESULTS

### 3.1 Comparative cohort descriptive characteristics

The total number of respondents to the survey was 232. Of the 232 participants, the majority were female (69%), aged between 18 and 25 years of age (40%), educated to degree level (49%), and had no religious faith (63%). There was an almost even split of participants that had children and those that did not (48% and 51.5%, respectively). Only 3% of respondents were either pregnant or trying to get pregnant. All participants had no relationship with SMA (Table 1).

**Table 1:** Participant demographics

Characteristic	General population (*n* = 232)
Gender – no. (%)	
Male	73 (31)
Female	159 (69)
Age (%)	
18–25 years	93 (40)
26–34 years	29 (12.5)
35–45 years	24 (10)
46–55 years	37 (16)
56–65 years	29 (12.5)
>66 years	20 (9)
Religious faith (%)	
Yes	74 (32)
No	146 (63)
Prefer not to say	12 (5)
Children (do you have) (%)	
Yes	111 (48)
No	120 (51.5)
Prefer not to say	1 (0.5)

### 3.2 Newborn Genetic Screening

Of the 232 people surveyed, 84% of participants were in favor of a newborn genetic screening program for SMA (Table 2). This is significantly higher than the views of people with SMA and their families where 70% of survey participants were in favor (*p* < .001). Most participants agreed that a newborn screening program would lead to better support and health care for the child with SMA and their family, it would help research into treatments by enabling more children to be enrolled into clinical trials early on and that it would enable parents to make informed decisions about future pregnancies (Table 2). It is important to note that 82% of the general population surveyed agreed that despite the inability to diagnose an SMA type, it is still important to have a diagnosis at birth (Table 2).

**Table 2:** A comparison of SMA families and the general population (views on newborn screening)

Question	GenPop (*n* = 232)	UK SMA population (AwS and Families; *n* = 337)	*p*‐value
Q1. Identifying SMA at birth would lead to better support for children and families (%)			
Agree	215 (93)	282 (84)	.001
Other	17 (7)	55 (16)	
Q2. Identifying SMA at birth would extend life expectancy of SMA children (%)			
Agree	118 (51)	127 (38)	.001
Other	114 (49)	210 (62)	
Q3. Identifying SMA at birth and not during pregnancy removes parents ability to make informed decisions about bringing SMA children into the world (%)			
Agree	145 (63)	192 (57)	.18
Other	87 (37)	145 (43)	
Q4. Identifying SMA before symptoms emerge will prevent families and children enjoying life while they are symptom‐free (%)			
Agree	60 (26)	149 (44)	<.0001
Other	172 (74)	188 (56)	
Q5. Identifying SMA at birth will help research by enabling more children to be enrolled into clinical trials early on (%)			
Agree	209 (90)	251 (74)	<.0001
Other	23 (10)	86 (26)	
Q6. Identification of SMA at birth would interfere with the early bonding process (%)			
Agree	30 (13)	50 (15)	.52
Other	202 (87)	287 (85)	
Q7. Identification of SMA at birth would make the diagnosis easier for parents to accept (%)			
Agree	118 (51)	100 (30)	<.0001
Other	114 (49)	237 (70)	
Q8. Identifying SMA at birth would spare the difficulties associated with finding a diagnosis for a child later on (%)			
Agree	185 (80)	222 (66)	.0003
Other	47 (20)	115 (34)	
Q9. Identifying SMA at birth is important, even if the Type cannot be determined (%)			
Agree	191 (82)	225 (67)	<.0001
Other	41 (18)	112 (33)	
Q10. Identifying SMA at birth is important because it will enable parents to make informed decisions about future pregnancies (%)			
Agree	217 (94)	272 (81)	<.0001
Other	15 (6)	65 (19)	
Q11. It is unethical to screen newborns for conditions that have no effective treatment (%)			
Agree	15 (6)	27 (8)	.48
Other	217 (94)	310 (92)	
Q12. I would support a Newborn screening program for SMA (%)			
Agree	196 (84)	236 (70)	<.0001
Other	36 (16)	101 (30)	

Of the questions asked regarding newborn screening, there were significant differences in the answers given by the general population and SMA families in nine of the 12 questions (Table 2). Arguably the most noticeable difference is that a significantly larger percentage of the general population surveyed agreed that identifying SMA at birth would make the diagnosis easier for the parents to accept. However, both groups agreed that identifying SMA at birth (and not in pregnancy) removes the parents’ ability to make informed decisions about bringing SMA children into the world, that it would not interfere with the early bonding process between parent and child and that it is not unethical to screen newborn babies for conditions which lack treatments (Table 2). The overall level of support for newborn genetic screening by the general population is similar to the overall support for other methods of SMA screening, namely preconception screening (86%) and prenatal screening (84%).

### 3.3 Why do the general population support newborn screening for SMA?

Univariate logistic regression shows that participants who were in support of a newborn genetic screening program for SMA believe that it will improve health care, extend the life expectancy of the child, improve research into treatments, spare difficulties associated with finding a diagnosis later on, and enable parents to make informed decisions about future pregnancies (Table 3). In addition, they agree that having a diagnosis at birth is important even if the particular type of SMA cannot be determined (Table 3). With regard to negative drivers, participants who supported a newborn screening program did not agree that identifying SMA at birth would prevent enjoyment of life while the child is still symptom‐free and that it would interfere with the early bonding process between parent and child. In addition, they did not agree that screening for conditions which have no effective treatments is unethical (Table 3).

**Table 3:** Univariate logistic regression showing positive and negative drivers of newborn screening support in general population

Question	Odds ratio (95% CI)	*p*‐value
Univariate logistic regression		
Q1. Identifying SMA at birth would lead to better support for children and families		
Other	Reference	<.0001
Agree	7.83 (2.79–22.04)	
Q2. Identifying SMA at birth would extend life expectancy of SMA children		
Other	Reference	.004
Agree	3.19 (1.46–6.97)	
Q3. Identifying SMA at birth and not during pregnancy removes parents ability to make informed decisions about bringing SMA children into the world		
Other	Reference	.09
Agree	184 (0.90–3.77)	
Q4. Identifying SMA before symptoms emerge will prevent families and children enjoying life while they are symptom‐free		
Other	Reference	.001
Agree	0.27 (0.13–0.57)	
Q5. Identifying SMA at birth will help research by enabling more children to be enrolled into clinical trials early on		
Other	Reference	.04
Agree	2.72 (1.03–7.17)
Q6. Identification of SMA at birth would interfere with the early bonding process		
Other	Reference	.001
Agree	0.24 (0.10–0.57)
Q7. Identification of SMA at birth would make the diagnosis easier for parents to accept		
Other	Reference	.4
Agree	1.36 (0.66–2.77)	
Q8. Identifying SMA at birth would spare the difficulties associated with finding a diagnosis for a child later on		
Other	Reference	.01
Agree	2.69 (1.24–5.84)	
Q9. Identifying SMA at birth is important, even if the Type cannot be determined		
Other	Reference	<.0001
Agree	5.47 (2.51–11.94)	
Q10. Identifying SMA at birth is important because it will enable parents to make informed decisions about future pregnancies		
Other	Reference	.0002
Agree	7.71 (2.60–22.93)	
Q11. It is unethical to screen newborns for conditions that have no effective treatment		
Other	Reference	.0002
Agree	0.13 (0.04–0.39)	

Multivariate logistic regression shows that those who lent support to newborn genetic screening did so because they believed it would lead to better support and healthcare and it would also enable parents to make informed decisions about future pregnancies. In addition, these participants believed that diagnosis was important even if the type of SMA could not be determined and they disagreed that it would interfere with the bonding process between the parents and the SMA child (Table 4). Inclusion of age, gender, qualification, and religious beliefs in the multivariate model did not alter the factors identified as independently associated with support, demonstrating there is no bias within the demographics of the respondents.

**Table 4:** Multivariate logistic regression showing most significant positive and negative drivers of newborn screening support in the general population

Multivariate logistic regression		
Question	Odds ratio (95% CI)	*p*‐value
Q1. Identifying SMA at birth would lead to better support for children and families		
Other	Reference	<.0001
Agree	6.07 (1.79–20.56)	
Q6. Identification of SMA at birth would interfere with the early bonding process		
Other	Reference	.004
Agree	0.24 (0.09–0.63)	
Q9. Identifying SMA at birth is important, even if the Type cannot be determined		
Other	Reference	.01
Agree	3.03 (1.23–7.46)	
Q10. Identifying SMA at birth is important because it will enable parents to make informed decisions about future pregnancies		
Other	Reference	.007
Agree	5.86 (1.61–21.32)	

## 4 DISCUSSION

This research was conducted in order to gather the general population's views on a newborn screening program for SMA in order to build on previous research that assessed their views on carrier screening (Boardman, 2017), and their contrast with the views of SMA families (Boardman et al., 2017a,b,c). Together, this research was carried out with the aim of assessing whether an SMA screening program would be acceptable to the general population, a key criterion when assessing the viability and feasibility of any new screening program in the UK (UK NSC, 2015).

Overall, the survey results demonstrate that 84% of participants from the general public were in favor of a newborn genetic screening program for SMA, in contrast to 70% support among SMA families (Table 3). The key reasons for screening support among the general population were a belief that it would lead to better support and healthcare and also that it would enable parents to make informed decisions about future pregnancies. In addition, the general public supported the notion that an early SMA diagnosis is important, irrespective of the ability to diagnose the specific type of SMA. This finding is significant as this limitation of the screening tests was considered a serious impediment to the introduction of SMA screening in the UK during the last policy review (Cartwright, 2012). It is noteworthy, therefore, that this issue was not considered a fatal flaw within the screening program by either the general population, or SMA‐affected families (Boardman et al., 2017a,b,c). For both groups, the importance of discovering SMA as early as possible outweighed the value of an accurate prognosis. In addition, the licensing of the first therapeutic for SMA in December 2016, Nusinersen, together with evidence that its efficacy is improved when administered to presymptomatic children (Bertini et al., 2017), is likely to further reinforce this viewpoint that earlier identification of the condition is critical.

The two key areas where members of the general population differed from SMA‐affected families in terms of screening support related to their acceptance of the diagnosis and the perceptions of the treatability of the condition (Boardman et al., 2017b). Within the general population sample, for example, there was a near 50/50 split between those who agreed that identifying SMA at birth would extend the life expectancy of the child (51% agree, 49% other), and those who did not. This number is significantly higher than among SMA families, for whom only 38% agreed that life expectancy could be extended through early diagnosis (Boardman et al., 2017b). This difference may not only highlight the disparity in condition‐specific knowledge and experience between the general population and affected families, but also points to the considerable cultural belief among the general population in the ability of medical interventions to alleviate, or even cure, chronic conditions like SMA. SMA families, however, many of whom had experienced the death of their child from SMA, or who experienced it as a chronic, untreatable condition were understandably more skeptical about the notion of cure (Boardman et al., 2017a) and as such were less likely to endorse this optimistic view that earlier identification and treatment necessarily leads to better outcomes for SMA children.

Another key area of divergence between the two populations related to the degree to which newborn screening was deemed to facilitate diagnosis acceptance. Members of the general population were considerably more likely to agree that an early diagnosis would make the condition easier for parents to accept than SMA families did (Boardman et al., 2017b). There is conflicting data within the literature on the impact of a diagnosis when it is received via a newborn screening program as opposed to after the onset of clinical symptoms. Some studies suggest that an earlier diagnosis, through screening, hastens the onset (and consequently the resolution) of parental grief, enabling them to more quickly come to terms with the condition and what it means for their family and future (Young & Tattersall, 2007). Other studies, however, have suggested that a diagnosis before a child is symptomatic can have a substantial negative impact on parental reactions. Grob (2008), for example, has argued that an unanticipated and unsolicited diagnosis may cause the early months of a child's life to become dominated by shock, anxiety and grief, which in turn interferes with the bonding process between parent and child (Grob, 2008). This reaction may indeed be exacerbated when the expected severity and life expectancy of the child remain uncertain, and the child may appear to be well and thriving at the point they are diagnosed with a serious‐ and potentially fatal‐genetic disorder. Indeed, in contrast to the general population, data from SMA families suggests that an SMA diagnosis is a difficult and painful experience for parents irrespective of when it is delivered (Boardman, 2010; Lawton, Hickerton, Archibald, McClaren, & Metcalfe, 2015), suggesting that earlier identification through newborn screening is unlikely to significantly reduce this impact.

Despite these two areas of contrast, however, the key reasons for supporting a newborn screening program appeared to be largely consistent between the general population and SMA families. This highlights that despite contrasting vantage points and experiences of the condition, both groups regarded early identification of SMA as the most significant benefit of newborn screening, even if an effective treatment was not available. This conviction underscores the importance of considering the experiences and viewpoints of the whole family when assessing the harms and benefits of newborn screening. For while, early identification in the absence of treatments may not have immediate and/or direct benefit to the child, the participants in this survey highlighted that there may, nevertheless, be considerable benefits to the family unit, including enhanced parental adjustment, acceptance of the condition, and future pregnancy planning.

When viewed in comparison to other forms of screening, the general population demonstrated the same level of support for a prenatal screening program as for a newborn one (84%). However, preconception screening was overall the most favored method of screening (86%) (Boardman et al., 2017a,b,c). This is in line with the views of SMA families who also favored preconception screening (77%), over prenatal (76%) and newborn screening (70%) (Boardman et al., 2017b,c). This suggests that although both groups agree that there are significant benefits to newborn screening, that the advantages of preconception screening (the possibility of avoiding the creation or carrying to term of SMA‐affected embryos/fetuses through early carrier identification) were viewed as more effective in tackling SMA than those associated with newborn screening, which instead focuses on treating infants who are already affected by SMA (Boardman, 2017; Boardman et al., 2017c).

Overall, this study highlights that the majority of the general population are in favor of newborn screening for SMA irrespective of the inability to diagnose the child with a particular type of SMA and despite the limited treatment options that are available. Newborn screening, unlike preconception and prenatal screening, does not potentially involve termination of pregnancy and for this reason is the least controversial screening method for SMA, particularly in the context of uncertain prognostic information. In addition, this screening method is the most likely to accelerate research into treatments by allowing early enrollment onto clinical trials, which is particularly important for conditions like SMA where symptom‐onset can be very early in life and the number of treatment options limited.

### 4.1 Limitations

Due to the anonymity of the survey link distributed, there was no way of preventing a respondent from completing the survey more than once. In addition, a high number of respondents were aged 18–25 years old (40%) and a significantly higher percentage of women responded to the survey than men (69% and 31% respectively). However, forward logistic regression taking into account gender, age, qualifications, and religion, showed no change in the main drivers behind support. Therefore, there appears to be no bias among the demographics of the respondents. Last, the final potential limitation could be the survey responder's limited knowledge of SMA. Although information on SMA was provided at the start of the survey in order to overcome this, some may argue that this information was not sufficient enough for the respondents to have a clear picture of the condition, or an understanding of what living with the condition on a day‐to‐day basis would be like. Similarly, it could be argued that the general population have not experienced the everyday difficulties associated with the condition and therefore have an altered perception of SMA which may affect the answers given. Nevertheless, responders were provided with background information before starting the survey and were free to pause the survey while they did their own independent research. They were then able to continue with the survey when they were happy they had gathered sufficient background knowledge of SMA and were therefore able to make informed decisions in order to answer the questions.

## CONFLICT OF INTEREST

All authors Felicity Boardman, Chloe Sadler, and Philip Young confirm that they have no conflicting interests to declare.
